# Motor Difficulties at 12 Years of Age Were Common in Children Born Very Preterm and Were Associated With ROP and Motor Performance in Infancy

**DOI:** 10.1111/apa.70681

**Published:** 2026-07-22

**Authors:** Cecilia Montgomery, Ylva Fredriksson Kaul, Martin Johansson, Sirkku Setänen, Kine Johansen, Katarina Strand Brodd, Lena Hellström‐Westas

**Affiliations:** ^1^ Department of Women's and Children's Health Uppsala University Uppsala Sweden; ^2^ Department of Psychology Uppsala University Uppsala Sweden; ^3^ Uppsala University Hospital Uppsala Sweden; ^4^ Department of Surgical Sciences, Neuroradiology Uppsala University Uppsala Sweden; ^5^ Pediatric Neurology, University of Turku and Turku University Hospital Turku Finland; ^6^ Centre for Clinical Research, Sörmland Uppsala University Uppsala Sweden

**Keywords:** brain injury, developmental coordination disorder, motor development, retinopathy of prematurity, very preterm born

## Abstract

**Aim:**

To examine motor competence at 12 years, and its association to motor performance in infancy, in children born very preterm without cerebral palsy.

**Methods:**

We prospectively assessed a regional cohort of 111 very preterm children, who were born in Sweden from 2004 to 2007 at 22–32 weeks of gestation. The Structured Observation of Motor Performance in Infancy was used to carry out motor assessments at four corrected ages to 12 months. At 12 years, 70 children without cerebral palsy were examined with the Movement Assessment Battery for Children (MABC‐2).

**Results:**

The mean total MABC‐2 score corresponded to the 16th percentile of the normative sample, and 16 children had results that indicated significant motor difficulties. Delayed motor development at 2 months and quality deficits at 10 months were related to motor difficulties at 12 years, after adjusting for gestational age and retinopathy of prematurity (ROP). Motor difficulties at 12 years were independently associated with ROP.

**Conclusion:**

Motor difficulties were prevalent in very preterm children, without cerebral palsy, at 12 years of age and were associated with poorer motor performance and ROP in infancy. The results highlight the importance of identifying motor difficulties early and continuing to monitor motor competence.

## Summary

- This study of 111 children born very preterm, without cerebral palsy, examined motor competence at 12 years of age and any association with motor performance in infancy.
- Average motor performance was below the norms at 12 years and almost a quarter of the 70 children assessed at this age had significant motor difficulties.
- Motor difficulties at 12 years were independently associated with atypical infant motor performance and retinopathy of prematurity.

**Abbreviations**
ADHD: attention deficit hyperactivity disorder
BPD: bronchopulmonary dysplasia
IVH: intraventricular haemorrhage
MABC‐2: the Movement Assessment Battery for Children, second edition
PVL: periventricular leukomalacia
ROP: retinopathy of prematurity
SOMP‐I: Structured Observation of Motor Performance in Infants

## 1 Introduction

Children born very preterm, below 32 weeks of gestation, have an increased risk of developing motor difficulties due to neonatal brain injuries and other factors that may disrupt normal brain development [1, 2]. This can cause both temporary and persisting motor problems [3, 4, 5]. Movement disorders, such as cerebral palsy and developmental coordination disorder, have been associated with delayed motor development in infants born very preterm [6, 7]. One study reported that cerebral palsy is typically diagnosed before 2 years of age [8]. Another found that motor difficulties often became apparent later, when children were expected to perform increasingly complex motor tasks [9]. Adequate motor competence, which includes the ability to perform skilled and coordinated movements, is important for daily functioning and school performance. It also affects physical and social activities, such as sports and play. Motor difficulties can limit a child's ability to fully engage in such activities and, this can negatively affect their social interactions, self‐esteem and overall development [5, 9, 10, 11].

Clinical follow‐up programs for children born very preterm aim to identify neurodevelopmental problems early and initiate training and support. However, many of these programs only focus on children at pre‐school or early school age [12], despite studies that have shown that motor competence may still be affected in adolescence. For example, a 2016 paper by Setänen et al. reported the results of a study of 98 Finnish children who had been born very preterm. At 11 years of age, 16% of the children had motor difficulties, which were defined as at or below the fifth percentile of the Movement Assessment Battery for Children, Second Edition (MABC‐2) [13]. Motor problems were associated with lower gestational age and bronchopulmonary dysplasia (BPD). A systematic review by Evensen et al. quantified the prevalence of motor difficulties in children born very preterm or with a very low birth weight and without cerebral palsy. These ranged from 8% to 37% at 5 years of age and older [4]. The same review identified a number of neonatal risk factors that were associated with motor outcomes, including male sex, lower gestational age, perinatal brain injuries, BPD and retinopathy of prematurity (ROP).

A number of studies have demonstrated associations between infant motor performance and later motor outcome in both full‐term and very preterm populations. However, most of these studies included children with cerebral palsy, focused on those born extremely preterm, and/or limited their follow‐up research to pre‐adolescence [14, 15, 16]. Investigating the relationship between motor competence in late childhood and motor performance in infancy may clarify the developmental trajectory of motor skills in children born very preterm without cerebral palsy. A better understanding of this relationship may facilitate earlier identification of those who could benefit from targeted support and underscore the importance of long‐term follow‐up to monitor and address evolving needs.

The aim of this study was to describe the motor competence of 12‐year‐old children born very preterm without cerebral palsy. We also wanted to examine any associations with motor performance during infancy in the light of relevant neonatal risk factors.

## 2 Methods

### 2.1 Participants

The prospective, population‐based Longitudinal Study of Visuomotor Capacity (LOVIS) was carried out at Uppsala University Hospital, Sweden, from 2004 to 2007 [17]. It focused on 113 very preterm infants, who were born at < 32 weeks of gestation. Two died after they were included in the study and neonatal characteristics were prospectively collected on the surviving 111, as previously described [18]. The neonatal risk factors examined by this study included sex, gestational age, birth weight and any degree of ROP. They also included severe brain injury, which was defined as grade 3–4 intraventricular haemorrhage and/or periventricular leukomalacia and BPD.

Motor performance in infancy was assessed using the Structured Observation of Motor Performance in Infants (SOMP‐I) at 2, 4, 6 and 10 months of corrected age. The number of participants at these ages varied between 89 and 101 [19].

At 12 years of age, 79/111 (72%) children participated in a follow‐up assessment that included evaluating motor competence using the MABC‐2 [20]. One child with postnatal brain injury was excluded from the present evaluation as well as the eight children who had been diagnosed with cerebral palsy. The early motor development of the children with cerebral palsy has already been reported previously [21]. Information on neurodevelopmental disorders, namely autism, attention deficit hyperactivity disorder (ADHD) and intellectual disability, was also collected at 12 years. Figure 1 describes the study participants who underwent assessments at 2, 4, 6 and 10 months of corrected age and 12 years.

**FIGURE 1:**
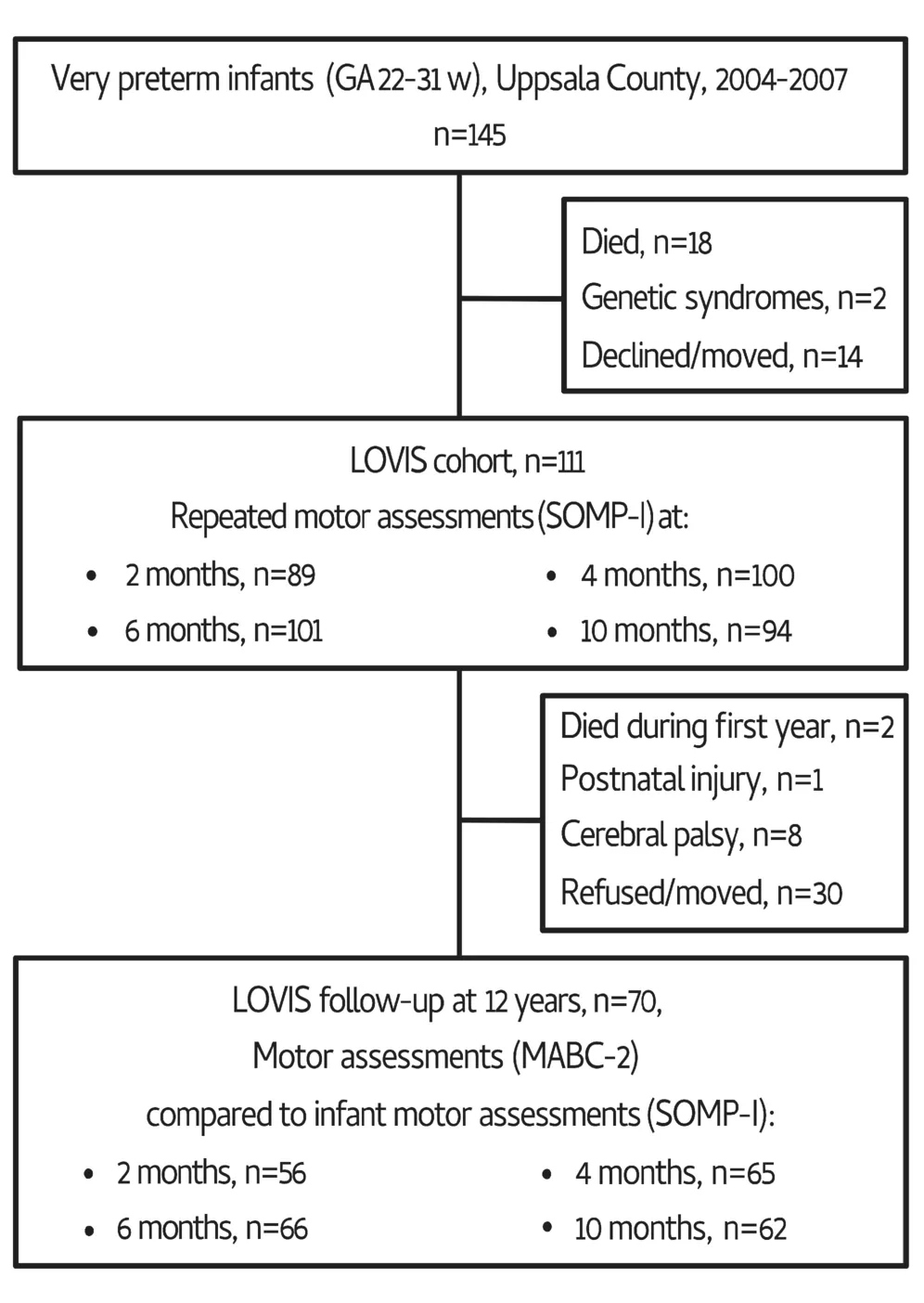
Flow chart of the participants with motor assessments from the LOVIS study group (Longitudinal study of Visuomotor capacity) at 2, 4, 6 and 10 months and 12 years of age.

### 2.2 Motor Assessment Methods

#### 2.2.1 MABC‐2

The motor assessments were performed with the MABC‐2 at 12 years of age [20]. This is suitable for children aged 11–16 years and includes three subscales: manual dexterity, aiming and catching and static and dynamic balance. Raw test scores were converted into subset and total standard test scores, with a mean of 10 ± 3 and presented as age‐adjusted percentile scores. Results above the 15th percentile indicated typical motor development, while results from the 6th to the 15th percentile suggested possible risks of motor difficulties. Scores up to the 5th percentile indicated significant motor difficulties [20]. UK norms were used as the reference material, because there were no Swedish norms [20].

The infant's motor performance was assessed using the SOMP‐I. This method is suitable for infants aged 0–12 months, and it evaluates two domains of motor function. These are the level of motor development and quality of motor performance [19]. The SOMP‐I provides a structured assessment of spontaneous and voluntary movements across 12 scales, covering supine and prone positions, sitting, standing, locomotion and hand function. The highest level of motor development that the infant achieved for each of the 12 scales was recorded. The maximum total score for the level of motor development was 72, with higher scores indicating more advanced development. Once the level had been determined, the quality of motor performance was assessed based on the predefined standards associated with that level. Quality was rated as adequate if there were no points, slight deficits if there was one point and pronounced deficits if there were two. The maximum total quality score was 24 [19, 22]. In contrast to those levels, a higher total score reflected greater deficits in the quality of motor performance.

The total scores for level and quality were interpreted using percentile distributions that were based on a reference group of 72 healthy full‐term neonates [23]. Scores of the 25th percentile or more were considered adequate, scores between the 6th and 24th percentiles indicated a slight delay, and scores of the 5th percentile or less indicated a pronounced delay. With regard to the quality of motor performance, scores up to the 75th percentile were considered adequate, scores between the 76th and 94th percentiles indicated slight deficits, and the 95th percentile or more indicated pronounced deficits.

The SOMP‐I examinations were performed at 2, 4, 6 and 10 months of corrected age. This meant that the number of assessments varied slightly between the four time‐points. Physiotherapists followed milder deviations in motor quality and/or milder delays in motor performance without any clinical suspicion of cerebral palsy. The parents were given training instructions. Infants with suspected or evident cerebral palsy were referred to paediatric neurologists for further evaluation and treatment and not included in the present study.

### 2.3 Ethics Statement

The Regional Ethics Board in Uppsala approved the study conducted during infancy (Dnr Ups 03‐665) and additional ethical approval was obtained for the 12‐year follow‐up tests (Dnr. 2016/400). Informed oral and written consent were obtained from the legal guardians.

### 2.4 Statistical Analysis

Continuous data were assessed for normality, and parametric Student's *t*‐test or non‐parametric Mann–Whitney *U* test were used as appropriate. Categorical variables were analysed using the chi‐square test or Fisher's exact test. Univariate and hierarchical multivariate logistic regression analyses were then conducted. The MABC‐2 used a cut‐off up to the 5th percentile to define the bivariate dependent variable. This indicated the presence or absence of significant motor difficulties for both total motor competence and the three subscales. Participant characteristics at 12 years of age were included as independent variables in the univariate analyses. We also included SOMP‐I percentile classifications for level, namely slight delay or pronounced delay, and quality, namely adequate, slight deficits or pronounced deficits. The potential neonatal risk factors that were examined were male sex, gestational age in weeks, birth weight, any degree of ROP, severe brain injuries and BPD. The level and quality at 2, 4, 6 and 10 months of corrected age were entered in Step 1 in separate models. Step 2 included two factors that showed significant univariate associations to motor competence at 12 years. These were gestational age and ROP, which was divided into no ROP or ROP stages 1–3. The number of children assessed at each time point during infancy varied slightly. To address this, we conducted drop‐out analyses to examine potential differences in neonatal characteristics between the participants and non‐participants at each age. Odds ratios (ORs) with 95% confidence intervals (CIs) are reported and the chi‐square difference test was used to evaluate improvements between the steps.

A two‐tailed *p*‐value of < 0.05 was considered statistically significant. All analyses were performed using SPSS version 27.0 (IBM Corporation, New York, USA).

## 3 Results

Table 1 presents the neonatal and 12‐year characteristics of the 70 subjects (54.3% male) in the study cohort. The univariate regression analyses showed that lower gestational age and any degree of ROP were significantly associated with motor outcomes up to the 5th percentile at 12 years of age. However, male sex, birth weight, brain injuries and BPD were not. Children with motor difficulties had a higher prevalence of neurodevelopmental conditions, namely autism, ADHD, and/or intellectual disabilities.

**TABLE 1:** Characteristics of the 12‐year‐old children who were born very preterm who did not have cerebral palsy and were assessed with the Movement Assessment Battery for Children Second edition (MABC‐2). Clinical characteristics are presented separately for children with significant motor difficulties (MABC‐2 ≤ 5th percentile) and those without (MABC‐2 > 5th percentile).

Characteristics	Study group	Motor difficulties yes/no		Odds ratio (95% CI)	*p*
	*N* = 70	≤ 5th percentile	> 5th percentile		
		*n* = 16	*n* = 54		
*Perinatal and neonatal factors*					
Sex (boys), *n* (%)	38 (54.3)	10 (62.5)	28 (51.8)	1.55 (0.44–4.86)	0.45
Gestational age (weeks), mean (SD)	28.6 (2.4)	27.4 (3.1)	29.0 (2.0)	0.75 (0.59–0.95)	0.02
Birth weight (g), mean (SD)	1191.9 (343.3)	1054.0 (393.8)	1232.7 (319.6)	0.998 (0.997–1.00)	0.07
Retinopathy of prematurity, stage 1–3^a^, *n* (%)	19 (27.1)	9 (56.3)	10 (18.5)	2.47 (1.41–4.29)	0.001
Severe brain injury^b^, *n* (%)	5 (7.1)	1 (6.2)	4 (7.4)	0.83 (0.09–8.03)	0.88
Bronchopulmonary dysplasia^c^, *n* (%)	16 (22.9)	6 (37.5)	10 (18.5)	2.64 (0.78–8.97)	0.12
*Neurodevelopmental outcome at 12 years*					
Autism spectrum disorder, *n* (%)	4 (5.7)	3 (18.8)	1 (1.9)		
Attention deficit hyperactivity disorder, *n* (%)	7 (10.0)	4 (25.0)	3 (5.6)		
Intellectual disability, *n* (%)	3 (4.3)	3 (18.8)	0 (0.0)		
One or more neurodevelopmental diagnosis, *n* (%)	11 (15.7)	7 (43.8)	4 (7.4)	0.66 (0.46–0.96)	0.03

^a^ Retinopathy of prematurity (stage 1: *n* = 1; stage 2: *n* = 15; stage 3: *n* = 3).

^b^ Presence of intraventricular haemorrhage, grade 3–4 and/or periventricular leukomalacia.

^c^ Bronchopulmonary dysplasia, severe = 30% supplementary oxygen at 36 weeks of gestational age.

The mean and standard deviation MABC‐2 total score for the 70 participating children was 66.8 ± 15.9, which corresponded to the 16th percentile of the normative sample (Table 2). We found that 16 children (23%) scored up to the 5th percentile, with a mean total score of 45.6 ± 11.3, which corresponded to the 2nd percentile. The remaining 54 children had a mean total score of 75.2 (7.5), which corresponded to the 37th percentile. Children with motor difficulties demonstrated the lowest performance on the manual dexterity and aiming and catching subscales, with mean scores that were equivalent to the 1st and 2nd percentiles, respectively (Table 2). The children without motor difficulties exhibited a more uniform motor profile across the subscales, with mean scores corresponding to the 37th percentile.

**TABLE 2:** MABC‐2 total score and subscale scores and corresponding percentile (pct) categories, based on the test manual.

MABC‐2 scores	Study group (*n* = 70)	pct	Motor difficulties, 12 years (*n* = 16)	pct	No motor difficulties, 12 years (*n* = 54)	pct
Mean (standard deviation)	Mean (SD)		Mean (SD)		Mean (SD)	
Total score	66.8 (15.9)	16	45.6 (11.3)	2	75.2 (7.5)	37
Manual dexterity (*n* = 14/56)	23.3 (8.1)	16	11.4 (5.4)	1	26.8 (4.6)	37
Aiming and catching (*n* = 20/50)	15.9 (5.5)	25	10.9 (4.0)	2	17.3 (5.0)	37
Balance (*n* = 7/63)	27.6 (6.6)	25	20.1 (6.2)	9	29.9 (4.8)	37

*Note:* Green: ≥ 15th percentile, typical motor development; Yellow: 6th–15th percentile, at risk for motor difficulties; Red: ≤ 5th percentile, significant motor difficulties.

The infants' motor performance, which was assessed using the SOMP‐I, is presented in Table 3. This shows the findings for the total study group and classifies the results by motor outcome at 12 years, by comparing up to, and above, the 5th percentile on the MABC‐2. All 70 children demonstrated slight delays in the level of motor development at 2 and 10 months, while the mean total scores at 4 and 6 months were within the adequate range. The mean total quality scores reflected adequate performance at a group level at all time points. There were 16 children with motor difficulties at 12 years, defined as up to the 5th percentile. Their mean SOMP‐I total scores indicated pronounced motor delays at 2 months, slight delays at 6 and 10 months and slight quality deficits at 4 and 10 months. In contrast, the 54 children with typical motor outcomes, above the 5th percentile, showed slight delays in the level of motor development at 2 and 10 months, but no quality deficits at any age.

**TABLE 3:** Motor outcomes at 12 years shown in relation to the group mean (SD) SOMP‐I total scores with corresponding percentile categories are shown in colour at 2, 4, 6 and 10 months of corrected age.

Infant motor performance	*N* = 70	Study group	*n* = 16	Motor difficulties at 12 years	*n* = 54	No motor difficulties at 12 years
				*n* = 16		*n* = 54
		Mean (SD)		Mean (SD)		Mean (SD)
*Months*	*Level of motor development*					
2	56	20.5 (3.4)	14	18.6 (2.7)	42	21.1 (3.4)
4	65	34.6 (4.0)	16	33.5 (4.8)	49	34.9 (3.6)
6	66	46.6 (5.4)	14	44.3 (6.8)	52	47.2 (4.9)
10	62	64.7 (5.2)	16	62.2 (6.2)	46	65.6 (4.6)
*Months*	*Quality of motor performance*					
2	56	5.2 (3.0)	14	4.4 (3.6)	42	5.4 (2.8)
4	65	5.7 (2.6)	16	6.3 (2.7)	49	5.6 (2.6)
6	66	5.4 (2.9)	14	6.6 (2.9)	52	5.1 (2.9)
10	62	2.3 (2.5)	16	3.6 (3.1)	46	1.9 (2.0)

*Note:* Green: adequate level (≥ 25th percentile) or adequate quality (≤ 75th percentile); Yellow: slight delay (≤ 24th–6th percentile) or slight deficits (76th–94th percentile) in quality; Red: pronounced delay (≤ 5th percentile) or pronounced deficits (≥ 95th percentile) in quality.

Delayed motor development at 2 months was significantly associated with motor difficulties at 12 years, when the unadjusted (*p* < 0.001) and adjusted models (*p* = 0.017) were controlled for gestational age and ROP (Table 4).

**TABLE 4:** Unadjusted and adjusted odds ratios (OR) for infant motor assessments (SOMP‐I) associated to motor difficulties at 12 years of age (MABC‐2) in children born very preterm without cerebral palsy.

Model	Step	Input variables at corrected ages	Chi‐square (df)	OR (95% CI)	*p*
1	1		5.85 (1)		0.016
		Level at 2 months		2.56 (1.13–5.76)	0.020
	2		13.81 (2)		< 0.001
		Level at 2 months		3.80 (1.27–11.34)	0.017
		Gestational age		1.17 (0.76–1.78)	0.901
		Retinopathy of prematurity		4.40 (1.45–13.46)	0.009
2	1		2.69 (1)		0.101
		Level at 4 months		1.98 (0.88–4.44)	0.098
	2		10.07 (2)		0.007
		Level at 4 months		1.96 (0.79–4.91)	0.147
		Gestational age		1.02 (0.71–1.47)	0.901
		Retinopathy of prematurity		2.41 (1.09–5.31)	0.029
3	1		3.75 (1)		0.053
		Level at 6 months		2.61 (0.97–7.06)	0.059
	2		11.40 (2)		0.003
		Level at 6 months		1.56 (0.47–5.13)	0.463
		Gestational age		0.89 (0.62–1.30)	0.554
		Retinopathy of prematurity		2.24 (0.99–5.05)	0.051
4	1		3.52 (1)		0.061
		Level at 10 months		2.25 (0.94–5.36)	0.067
	2		9.34 (2)		0.009
		Level at 10 months		2.26 (0.84–6.05)	0.104
		Gestational age		1.09 (0.75–1.58)	0.655
		Retinopathy of prematurity		2.61 (1.15–5.93)	0.022
5	1		0.57 (1)		0.449
		Quality 2 months		0.63 (0.18–2.18)	0.46
	2		11.68 (2)		0.003
		Quality 2 months		1.17 (0.28–4.91)	0.830
		Gestational age		1.04 (0.70–1.53)	0.858
		Retinopathy of prematurity		2.88 (1.15–7.19)	0.023
6	1		1.79 (1)		0.182
		Quality 4 months		1.85 (0.75–4.58)	0.190
	2		12.25 (2)		0.002
		Quality 4 months		2.72 (0.92–8.12)	0.072
		Gestational age		1.04 (0.73–1.49)	0.818
		Retinopathy of prematurity		2.88 (1.19–6.95)	0.018
7	1		4.09 (1)		0.043
		Quality 6 months		2.62 (1.02–6.78)	0.046
	2		13.17 (2)		0.001
		Quality 6 months		2.53 (0.81–7.91)	0.111
		Gestational age		0.88 (0.60–1.30)	0.532
		Retinopathy of prematurity		2.41 (1.03–5.66)	0.043
8	1		6.01 (1)		0.014
		Quality 10 months		2.68 (1.29–6.01)	0.020
	2		10.11 (2)		0.006
		Quality 10 months		3.05 (1.22–7.64)	0.017
		Gestational age		0.93 (0.70–1.49)	0.927
		Retinopathy of prematurity		2.55 (1.09–5.94)	0.031

*Note:* Hierarchical logistic regression analyses. Models 1–4 examine the level of motor development at 2, 4, 6 and 10‐months of corrected age, while Models 5–8 examine the quality of motor performance at the same ages. In all models, Step 1 includes the three SOMP‐I percentile categories (adequate, slight delay/deficit, pronounced delay/deficit), with motor difficulties (MABC‐2 ≤ 5th percentile) as the dependent variable. Step 2 adds gestational age and retinopathy of prematurity (ROP) as covariates. Chi‐square (df) indicates the improvement in model fit between steps. Df stands for degrees of freedom.

Poor quality motor performance at 10 months was significantly associated with increased odds of later motor difficulties in both the unadjusted (*p* = 0.006) and adjusted (*p* = 0.017) (Table 4).

These models showed that ROP made unique contributions to motor outcome. When we carried out a sensitivity analysis that excluded the three children with severe ROP, defined as stage 3, the associations were attenuated, but remained statistically significant (OR 2.04, 95% CI 1.06–3.9, *p* = 0.03).

Additional associations between infant motor performance and the MABC‐2 subscale scores are presented in Table S1. At 2 months, poor quality was associated with lower performance in aiming and catching (*p* = 0.048). Delayed levels at six (*p* = 0.02) and 10 months (*p* = 0.003) and quality deficits at 10 months (*p* = 0.005) were associated with poorer balance scores at 12 years.

Drop‐out analyses showed that at 2 months, 56 of the infants that were included in the follow‐up assessments at 12 years had a lower prevalence of periventricular leukomalacia than the 14 who had not taken part (*p* = 0.02). At 4 months, a higher proportion of examined participants were male than children who did not participate at 12 years of age (58.5% vs. 54.3%) (*p* = 0.02) (Table S2). No other significant differences in neonatal characteristics were observed at the various infant assessment ages.

Table S3 presents the neonatal risk factors related to the MABC‐2 subscales. ROP was significantly associated with a lower score on all subscales: manual dexterity (*p* = 0.02), ball skills (*p* = 0.005) and balance (*p* = 0.04). Lower gestational age was associated with poor performance on the aiming and catching scale (*p* = 0.045) and the balance scale (*p* = 0.03). Boys achieved significantly higher total scores than girls on aiming and catching (*p* = 0.045), whereas BPD was negatively associated with performance on the balance subscale (*p* = 0.04).

## 4 Discussion

This population‐based study showed that the motor competence of 12‐year‐old children who were born very preterm, without cerebral palsy, was substantially below average. The group mean close to the cut‐off was used to define the risk for motor difficulties in a normative population. Furthermore, almost a quarter of the children scored up to the 5th percentile on the MABC‐2, which indicated that they had significant motor difficulties. The SOMP‐I showed that some of these children were repeatedly, but not consistently, categorised as having poorer motor performance, with delayed levels and/or quality deficits. ROP was independently associated with motor difficulties at 12 years of age.

The average motor performance of the preterm group was below the normative mean at 12 years of age. This suggests that children born very preterm may have poorer motor abilities than their full‐term peers, regardless of other diagnoses. The impact that motor difficulties have on participation, quality of life and the risks of secondary challenges, such as obesity and depression, is already well known. Our findings support the need to extend neurodevelopmental follow‐up visits for children born very preterm into adolescence [9, 10, 24].

Almost half of the children who exhibited motor difficulties had at least one neurodevelopmental disorder. This may have reflected the higher prevalence of conditions, such as ADHD, autism and intellectual disability, among very preterm children. It may have also been due to the frequent co‐occurrence of motor difficulties with these diagnoses [9, 25]. The frequent co‐occurrence of motor difficulties and neurodevelopmental disorders warrants the need for motor assessments to be included in neurodevelopmental evaluations, especially when children were born very preterm. This will enable such children to receive appropriate support.

The association between motor performance in infancy and motor competence at 12 years of age was not consistent at all ages. This suggests that early motor difficulties do not invariably predict long‐term outcomes. A systematic review published in 2021 investigated the predictive ability of pre‐school motor examinations for later motor difficulties. The review showed a number of infant assessment methods predicted later motor problems, but none of them reached a specificity and/or sensitivity of more than 80% for identifying motor difficulties at school age. Motor assessments conducted after infancy tended to have higher predictive values [26]. This may be explained by greater variations in early motor development, which were characterised by periods of rapid progress followed by plateaus [27]. In addition, early assessment tools may have lacked sufficient sensitivity to identify motor difficulties other than cerebral palsy. It is therefore particularly noteworthy that our study found that motor performance at 2 and 10 months of corrected age was associated with motor competence at 12 years. This could have been due to the fact that we assessed two separate domains, namely level and quality.

However, the inconsistent findings suggest a more complex developmental trajectory. Motor difficulties may become more apparent as motor abilities evolve and the demand on tasks increases with age. This may indicate that early markers may not fully capture the progression of motor challenges. Although early motor support is essential, it should be combined with long‐term follow‐up visits to optimise outcomes for this population.

It has been shown that neurodevelopment in very preterm children has been strongly influenced by perinatal risk factors [3]. Our study found that early motor performance and ROP each explained unique variances in 12‐year motor outcomes. Subscale analyses indicated that children with motor difficulties primarily struggled with hand function and aiming and catching. These rely heavily on adequate eye‐hand coordination. The association between ROP and motor difficulties may have reflected a shared underlying effect on the brain, as white matter injuries have been closely linked to ROP [28]. In addition, it has been shown that early visual impairment can disrupt visual‐motor integration [29].

### 4.1 Strength and Limitations

A key strength of the study was that we carried out repeated assessments of infant motor development. The method we used captured multiple dimensions of early motor functioning by assessing motor competence at 12 years while also accounting for neonatal risk factors.

A limitation of this study was that we lost some infants to follow up at various motor assessment points. Restricting the analyses to subjects with complete data from all four infant assessments would have substantially reduced the statistical power of our study. However, the neonatal characteristics were comparable at these ages, and this supported the validity of the analyses. We chose not to impute the missing data, as the inherently variable nature of early motor development could have obscured meaningful nuances.

UK norms were used for the 12‐year assessments, despite the availability of a term‐born control group. Using external norms risked underestimating or overestimating the proportion of children with motor difficulties [30]. Although this approach had limitations, it was chosen to enable us to compare our findings with other studies.

Another limitation was the relatively small sample size, as the parents of 30 of the 109 eligible participants declined participation or had moved away at the time of the 12‐year assessment. This particularly affected the subgroup with motor difficulties and limited the statistical power to detect more subtle associations.

## 5 Conclusion

This study demonstrated that children who were born very preterm, but without cerebral palsy, still faced an increased risk of motor difficulties at 12 years of age. Early motor delays and deficits in quality were associated with poorer motor outcomes but were not consistent across all assessments. The findings also suggest that there was a clear association between ROP in infancy and later motor competence. These combined results highlight the need for ongoing monitoring of motor competence in children born very preterm.

## Author Contributions

**Cecilia Montgomery:** writing – original draft, formal analysis, conceptualization, investigation, data curation. **Martin Johansson:** formal analysis, writing – review and editing, investigation, conceptualization, data curation. **Ylva Fredriksson Kaul:** conceptualization, investigation, writing – review and editing, data curation, visualization, formal analysis. **Katarina Strand Brodd:** conceptualization, writing – review and editing, supervision. **Kine Johansen:** writing – review and editing. **Sirkku Setänen:** investigation, writing – review and editing. **Lena Hellström‐Westas:** writing – review and editing, conceptualization, funding acquisition, project administration, supervision.

## Funding

This work was supported by the Swedish Research Council grant (Vetenskapsrådetin Swedish) (2016‐03109), the Promobilia Foundation, the Folke Bernadotte Foundation and Uppsala University and the Uppsala County Council Grants for Healthcare Research.

## Conflicts of Interest

The SOMP‐I method is the intellectual property of Movement Inspires AB, a company founded and operated by Kine Johansen. The other authors have no conflicts of interest to declare.

## Supporting information


**Table S1:** Unadjusted odds ratios (OR) for infant motor performance (SOMP‐I) in relation to MABC‐2 subscales at 12 years of age in children born very preterm without cerebral palsy.
**Table S2:** Drop‐out analysis comparing children who participated versus those who did not participate in the 12‐year follow‐up, presented separately for each infant assessment age.
**Table S3:** Characteristics of 12‐year‐old children born very preterm, assessed with the Movement Assessment Battery for Children Second edition (MABC‐2). Clinical characteristics are presented separately for children with significant motor difficulties (MABC‐2 **≤** 5th percentile) and those with typical motor development (MABC‐2 > 5th percentile), based on each of the three subscales: Manual Dexterity, Ball skills and Balance.

## Data Availability

The data that support the findings of this study are available from the corresponding author upon reasonable request.
