# Supplementary material for: Motor Difficulties at 12 Years of Age Were Common in Children Born Very Preterm and Were Associated With ROP and Motor Performance in Infancy
Source: Acta Paediatr. 2026 Jul 22;115(12):2765–73. doi: 10.1111/apa.70681 (PMC13747688; doi:10.1111/apa.70681)
Supplement: Supplementary file 1 [file APA-115-2765-s001.docx]

Supplementary table 1. Unadjusted odds ratios (OR) for infant motor performance (SOMP-I) in relation to MABC-2 subscales at 12 years of age in children born very preterm without cerebral palsy.

|  | **Manual dexterity** | | | | **Aiming and catching** | | | | **Balance, static and dynamic** | | | |
| --- | --- | --- | --- | --- | --- | --- | --- | --- | --- | --- | --- | --- |
|  | **≤ 5th**  **n=14** | **> 5th**  **n= 56** | **Odds ratio**  **(CI 95%)** | ***p*-value** | **≤ 5th**  **n=20** | **> 5th**  **n= 50** | **Odds ratio**  **(CI 95%)** | ***p*-value** | **≤ 5th**  **n=7** | **> 5th**  **n= 63** | **Odds ratio**  **(CI 95%)** | ***p*-value** |
| **Infant motor**  **performance**  **Mean (SD)** |  |  |  |  |  |  |  |  |  |  |  |  |
| ***2 months****, n* | 12 | 44 |  |  | 17 | 39 |  |  | 6 | 50 |  |  |
| Level | 18.8 (2.9) | 20.9 (3.4) | 0.82 (0.66-1.01) | 0.06 | 20.5 (3.5) | 20.5 (3.4) | 1.00 (0.85-1.20) | 0.92 | 19.3 (2.4) | 20.6 (3.5) | 0.89 (0.68-1.16) | 0.38 |
| Quality | 4.6 (3.6) | 5.3 (2.8) | 0.92 (0.73-1.14) | 0.45 | 3.9 (3.2) | 5.7 (2.8) | 0.80 (0.64-0.998) | 0.048 | 4.0 (3.6) | 5.3 (2.9) | 0.85 (0.62-1.17 | 0.32 |
| ***4 months****, n* | 14 | 51 |  |  | 18 | 47 |  |  | 7 | 58 |  |  |
| Level | 34.3 (4.7) | 34.7 (3.8) | 0.98 (0.84-1.13) | 0.77 | 33.8 (4.8) | 34.9 (3.6) | 0.94 (0.81-1.08) | 0.35 | 32.7 (3.3) | 34.8 (4.0) | 0.87 (0.71-1.07) | 0.19 |
| Quality | 6.3 (2.9) | 5.6 (2.6) | 1.12 (0.89, 1,41) | 0.33 | 5.8 (2.4) | 5.7 (2.7) | 1.00 (0.82-1.24) | 0.97 | 5.8 (2.7) | 5.3 (2.6) | 0.92 (0.68-1.25) | 0.61 |
| ***6 months****, n* | 12 | 54 |  |  | 17 | 49 |  |  | 7 | 59 |  |  |
| Level | 44.7 (7.3) | 47.0 (4.9) | 0.92 (0.81-1.04) | 0.20 | 45.2 (5.3) | 47.1 (5.5) | 0.93 (0.84-1.04) | 0.22 | 41.6 (3.5) | 47.2 (6.3) | 0.77 (0.62-0.96) | 0.02 |
| Quality | 6.2 (2.9) | 5.2 (2.9) | 1.14 (0.91-1.42) | 0.25 | 5.6 (3.1) | 5.3 (2.9) | 1.04 (0.86-1.26) | 0.70 | 7.5 (1.9) | 5.2 (2.9) | 1.36 (0.99-1.88) | 0.06 |
| ***10 months****, n* | 14 | 48 |  |  | 17 | 45 |  |  | 7 | 55 |  |  |
| Level | 62.2 (6.2) | 65.4 (4.7) | 0.89 (0.80-1,00) | 0.05 | 62.6 (6.4) | 65.5 (4.5) | 0.90 (0.81-1.00) | 0.06 | 57.6 (5.7) | 65.2 (4.4) | 0.76 (0.63-0.91) | 0.003 |
| Quality | 3.9 (3.3) | 1.9 (2.0) | 1.34 (1.06-1.71) | 0.01 | 3.1 (3.3) | 2.0 (2.0) | 1.20 (0.97-1.50) | 0.09 | 5.2 (3.8) | 1.9 (2.0) | 1.54 (1.14-2.10=) | 0.005 |

Supplementary table 2. Drop-out analysis comparing children who participated versus those who did not participate in the 12-year follow-up, presented separately for each infant assessment age.

| **Characteristics** |  | **Infant assessments ages** | | | |
| --- | --- | --- | --- | --- | --- |
|  | **Study group** | **2 months** | **4 months** | **6 months** | **10 months** |
|  | N=70 | n=56 | n=65 | n=66 | n=62 |
| ***Perinatal and neonatal factors*** |  |  |  |  |  |
| Sex (boys), n (%) | 38 (54.3) | 28 (50.0) | 38 (58.5) | 36 (54.5) | 36 (58.1) |
| Gestational age (weeks), mean (SD) | 28.6 (2.4) | 27.9 (2.5) | 28.1 (2.4) | 28.12 (2.4) | 28.0 (2.4) |
| Birth weight (g), mean (SD) | 1192 (343) | 1172 (353) | 1183 (348) | 1184 (345) | 1184 (350) |
| Retinopathy of prematurity^a^, n (%) | 19 (27.1) | 18 (32.1) | 19 (29.2) | 19 (28.8) | 18 (29.0) |
| Intraventricular hemorrhage, grade 3-4, n (%) | 2 (2.9) | 1 (1.8) | 2 (3.1) | 2 (3.0) | 2 (3.2) |
| Periventricular leukomalacia, n (%) | 4 (5.7) | 1 (1.8) | 3 (4.6) | 4 (6.1) | 3 (4.8) |
| Severe brain injury^b^ | 5 (7.1) | 2 (3.6) | 4 (6.2) | 5 (7.6) | 4 (6.5) |
| Bronchopulmonary dysplasia^c^, n (%) | 16 (22.9) | 12 (21.4) | 16 (24.6) | 16 (24.2) | 15 (24.2) |
| ***Follow-up characteristics at 12 years*** |  |  |  |  |  |
| Autism spectrum disorder, n (%) | 4 (5.7) | 3 (5.4) | 4 (6.2) | 3 (4.5) | 4 (6.5) |
| Attention deficit hyperactivity disorder, n (%) | 7 (10.0) | 6 (10.7) | 6 (9.2) | 6 (9.1) | 7 (11.3) |
| Intellectual disability | 3 (4.3) |  |  |  |  |
| ***Motor competence at 12 years*** |  |  |  |  |  |
| MABC-2 total score, mean (SD) | 66.8 (15.9) | 65.5 (15.9) | 66.4 (16.4) | 67.4 (15.6) | 65.8 (16.4) |

^a^Retinopathy of prematurity (stage 1: n=1; stage 2: n=15; stage 3: n=3)

^b^Presence of both intraventricular hemorrhage, grade 3-4 and/or periventricular leukomalacia

^c^Bronchopulmonary dysplasia, severe 30% supplementary oxygen at 36 weeks of gestational age.

Supplementary table 3. Characteristics of 12-year-old children born very preterm, assessed with the Movement Assessment Battery for Children Second edition (MABC-2). Clinical characteristics are presented separately for children with significant motor difficulties (MABC-2 **≤** 5th percentile) and those with typical motor development (MABC-2 >5^th^ percentile), based on each of the three subscales: Manual Dexterity, Ball skills and Balance.

|  | **Manual dexterity** | | | | **Aiming and catching** | | | | **Balance, static and dynamic** | | | |
| --- | --- | --- | --- | --- | --- | --- | --- | --- | --- | --- | --- | --- |
|  | ≤ 5th  n=14 | > 5th  n= 56 | Odds ratio  (CI 95%) | *p*-value | ≤ 5th  n=20 | > 5th  n= 50 | Odds ratio  (CI 95%) | *p*-value | ≤ 5th  n=7 | > 5th  n= 63 | Odds ratio  (CI 95%) | *p*-value |
| ***Perinatal and neonatal factors*** |  |  |  |  |  |  |  |  |  |  |  |  |
| Sex (boys), n (%) | 9 (64.3) | 29 (51.8) | 1.68  (0.50-5.63) | 0.40 | 7 (35.0) | 31 (62.0) | 0.33 (0.11-0.97) | 0.045 | 4 (57.1) | 34 (54.1) | 1.14  (0.24-5.50) | 0.87 |
| Gestational age (weeks), mean (SD) | 28.0 (3.0) | 28.7 (2.2) | 0.88  (0.69-1.12) | 0.30 | 27.7 (2.7) | 29.0 (2.1) | 0.80 (0.64-1.00) | 0.045 | 26.7 (3.3) | 28.8 (2.2) | 0.71  (0.51-0.97) | 0.03 |
| Birth weight (g), mean ± SD | 1120.8  (408.8) | 1209 .6 (326.7) | 1.0  (1.0-1.0) | 0.39 | 1016.5 (326.2) | 1262.0  (327.3) | 0.998 (0.996-0.999) | 0.009 | 1038.6 (488.7) | 1208.9 (324.2) | 0.998 (0.996-1.001) | 0.22 |
| Retinopathy of prematurity^a^, n (%) | 6 (42.8) | 13 (23.2) | 1.82  (1.09-3.05) | 0.02 | 10 (50.0) | 9 (18.0) | 2.07 (1.24-3.45) | 0.005 | 3 (42.9) | 16 (26.7) | 1.89  (1.02-3.51) | 0.04 |
| Severe brain injury^b^, n (%) | 1 (7.1) | 4 (7.1) | 1.00  (0.10-9.71) | 1.00 | 1 (5.0) | 4 (8.0) | 0.61 (0.06-5.78) | 0.66 | 1 (14.3) | 4 (6.3) | 2.46  (0.24-25.7) | 0.45 |
| Bronchopulmonary dysplasia^c^, n (%) | 6 (42.8) | 11 (19.6) | 2.27  (0.63-8.14) | 0.21 | 7 (35) | 9 (18) | 2.45  (0.76-7.89) | 0.13 | 4 (57.1) | 12 (19.0) | 5.67  (1.12-28.73) | 0.04 |

^a^Retinopathy of prematurity (stage 1: n=1; stage 2: n=15; stage 3: n=3)

^b^Presence of intraventricular hemorrhage, grade 3-4 and/or periventricular leukomalacia

^c^Bronchopulmonary dysplasia, severe 30% supplementary oxygen at 36 weeks of gestational age.
